# Association of Weight Loss With Improved Sexual Function in Females

**DOI:** 10.7759/cureus.16849

**Published:** 2021-08-03

**Authors:** Asghar Hussain Syed, Aakash Chandnani, Asim Khan, Naila S Bhutto, Hamza Tahir, Abbas Iqbal, Suraj K Aruwani, Sidra Naz, Parkash Bachani

**Affiliations:** 1 Cardiology, National Institute of Cardiovascular Diseases, Karachi, PAK; 2 Internal Medicine, Jinnah Sindh Medical University, Karachi, PAK; 3 Internal Medicine, Lahore Medical and Dental College, Lahore, PAK; 4 Internal Medicine, Chandka Medical College, Larkana, PAK; 5 Internal Medicine, Allama Iqbal Medical College, Lahore, PAK; 6 Internal Medicine, Ayub Teaching Hospital, Abottabad, PAK; 7 Internal Medicine, University of Health Sciences, Lahore, PAK; 8 Internal Medicine, Liaquat University of Medical and Health Sciences, Jamshoro, PAK

**Keywords:** weight loss, sexual performance, fsfi score, obesity, libido

## Abstract

Introduction

The prevalence of obesity in developing countries, including Pakistan, has increased several fold in recent times. Obesity appears to negatively affect sexual functioning, hence affecting the quality of life. Its impact on sexual function is understudied. In this study, we will determine the impact of weight loss in improving sexual function in the local setting.

Methods

This prospective study was conducted in the endocrinology unit of a tertiary care hospital in Pakistan from February 2019 to January 2021. After taking informed consent, 300 married female participants were enrolled in the study. The questionnaire was composed using the pointers from the female sexual function index (FSFI). The privacy of the participants was fully ensured. After the survey, participants were counseled on losing weight via various techniques. Participants were followed up on day 30, day 60, and finally on day 90. On day 90, the FSFI questionnaire was repeated to assess sexual function. Weight loss was measured at the end of day 90.

Result

A total of 208 participants completed the study. Significant improvement in FSFI score was seen in participants with weight loss between 2% and 5% of their initial body weight (24.01 ± 2.2 vs. 26.07 ± 2.6; p-value: <0.0001). Similarly, a significant improvement in FSFI score was seen in participants with weight loss of more than 5% (24.17 ± 2.2 vs. 27.01 ± 2.6; p-value: <0.0001).

Conclusion

In conclusion, weight loss is associated with improved sexual function in females. While discussing complications of obesity, impact on sexual function should also be discussed.

## Introduction

The prevalence of obesity in developing countries, including Pakistan, has increased several fold. As per the global disease estimate, Pakistan is ranked eighth among 10 countries with the highest prevalence of obesity, according to a global burden study published in 2013 [1]. According to a study conducted in 2020, the prevalence of obesity in Pakistan is around 22% [2]. It is associated with various complications, such as type 2 diabetes, cardiovascular ailments, joint and mobility issues, respiratory issues like asthma, and psychological issues like anxiety and depression [3].

The impact of obesity on sexual function is understudied. However, few studies that are available indicate that obesity appears to negatively affect sexual functioning [4-6], hence affecting the quality of life. It has been hypothesized to cause erectile dysfunction in men [5], and weight reduction has been seen to show an improvement in sexual function. Kostis et al. reported significant improvements in sexual function in participants who lost approximately 5% of their weight through a lifestyle modification program [7].

There are very limited data on the impact of weight loss in improving sexual function, particularly from the South Asian region. In this study, we will determine the impact of weight loss in improving sexual function in the local setting.

## Materials and methods

This prospective study was conducted in the endocrinology unit of a tertiary-care hospital in Pakistan from February 2019 to January 2021. After taking informed consent, 300 married female participants, in the age group of 25-40 years and a body mass index (BMI) of more than 25 kg/m^2^, were enrolled in the study. Participants were enrolled via consecutive convenient non-probability sampling and were informed that they could withdraw their consent at any time of the study. Ethical review board approval was taken before the enrollment of participants. Participants with current smoking status, hypertension, diabetes, and polycystic ovarian disease were excluded from the study.

After enrollment, participants' weights were taken via digital scale and were noted in the questionnaire. The questionnaire was composed using the pointers from the female sexual function index (FSFI). A separate area was designated for the interview of the participants so that their privacy was fully ensured. The FSFI is a brief, multidimensional self-report instrument comprising 19 questions for assessing key dimensions of sexual function in women. It evaluates six domains of sexual functions, namely desire, arousal, lubrication, orgasm, satisfaction, and pain. The first two questions of the FSFI questionnaire have a scoring scale from one to five and the rest of the questions’ scores range from zero to five. The scores acquired are added up and multiplied by a respective factor (coefficients for questions 1, 2: 0.6, 3-10: 0.3, 11-19: 0.4). The scoring scale ranges from 1.2 to 36. A score less than 26 indicated poor sexual function while a higher score indicated a healthy sexual life [5,8]. 

After the survey, participants were counseled on losing weight. Participants were given personalized diet charts based on their body type. Along with the diet plan, various exercises were demonstrated to the participants to aid weight loss. Participants were asked to come for follow-up on day 30, day 60, and thereafter on day 90. On day 90, the FSFI questionnaire was repeated to assess the sexual function. Weight loss was measured at the end of day 90. On the basis of weight on day 0, weight loss was divided into three categories, i.e. no change or less than 2% weight loss, weight loss between 2% and 5%, and weight loss more than 5%.

We lost 92 participants to follow up. Only those participants who completed the final follow-up were included in the statistical analysis. Data analysis was done using the Statistical Package for Social Sciences, version 22.0 (SPSS, IBM Corporation, Armonk, New York, United States). Continuous variables were presented as mean and standard deviation. Percentages and frequencies were calculated for categorical variables. For the comparison of categorical data pre- and post-weight loss for each group, chi-square and dependent t-test were applied, as appropriate. A p-value of less than 0.05 was considered statistically significant.

## Results

A total of 208 participants completed the study. The mean FSFI score at day 0 was 24.16 ± 2.3 and 26.17 ± 2.6 at day 90 (Table 1).

**Table 1:** Characteristics of participants BMI: body mass index; FSFI: female sexual function index

Characteristics	Number
Age in years at the time of enrollment (mean ± SD)	31 ± 4
Mean BMI at the time of enrollment (kg/m^2^)	27.2 ± 2.3
Mean FSFI score	
Day 0	24.16 ± 2.3
Day 90	26.17 ± 2.6

A total of 208 participants completed the study. On day 0, 116 participants had an FSFI score of less than 26, compared to 83 participants on day 90. Significant improvement in FSFI score was seen in participants with weight loss between 2% and 5% of their initial body weight (24.01 ± 2.2 vs. 26.07 ± 2.6; p-value: <0.0001). Similarly, a significant improvement in FSFI score was seen in the participants with weight loss of more than 5% (24.17 ± 2.2 vs. 27.01 ± 2.6; p-value: <0.0001) (Table 2).

**Table 2:** Assessment of FSFI score in association with weight changes FSFI: female sexual function index; SD: standard deviation

Weight change (n = 208)	FSFI score	Day 0	Day 90	p-Value
No weight loss or less than 2% (n = 62)	Total sexual score (mean ± SD)	25.01 ± 2.9	25.12 ± 2.7	0.82
	Participants with score less than 26	35 (56.4%)	37 (59.6%)	0.58
Weight loss between 2% and 5% (n = 77)	Total sexual score (mean ± SD)	24.01 ± 2.2	26.07 ± 2.6	<0.0001
	Participants with score less than 26	41 (53.3%)	25 (32.4%)	0.009
Weight loss more than 5% (n = 69)	Total sexual score (mean ± SD)	24.17 ± 2.2	27.01 ± 2.6	<0.0001
	Participants with score less than 26	40 (57.9%)	21 (30.4%)	0.0011

## Discussion

In this study, weight loss was associated with a significant improvement in sexual function. This improvement was seen in groups with weight loss between 2% and 5% and more than 5% of initial body weight. There was no change in sexual function in participants who lost none to very negligible weight.

In concordance with the findings of our study, several other studies have also proved the link between weight loss and improved sexual function. A study including a sample size of more than 300 obese women, having a mean age of 53 years, analyzed the impact on the reduction of weight by measures like dietary group meetings, calculated calorie use, appetite suppressant (sibutramine), and regular exercise. Women whose weight reduced demonstrated betterment in arousal and orgasm [5]. In another study, Aversa et al. studied 44 women to check the effects of dietary measures, exercise, and weight loss. It was observed that reduced weight led to a considerable enhancement in sexual arousal, lubrication, and sexual satisfaction [8]. This study also concluded that weight loss could potentially improve endothelial function and insulin resistance, which relieve the factors that cause sexual dysfunction in women. Another study with similar objectives involving 200 obese sexually active women concluded that 48.3% had desire-related problems, 35.9% with arousal, 45.0% with lubrication, and 42.9% experienced pain during sex [9].

There are several theories that explain the link between obesity and its effect on sexual function. It is a known fact that obesity puts individuals at a higher risk of cardiometabolic diseases that in turn are linked with reduced sexual activity, like type 2 diabetes, dyslipidemia, and hypertension [10,11]. Endothelial function and genital blood flow also form a close link between cardiometabolic health and sexual function, but sufficient evidence supporting this link is not available [12-16]. Moreover, the effect of metabolic syndrome on increased sexual function is controversial [17,18]. Obese people are more likely to report problems of anxiety and depression; these factors are believed to have a direct or indirect connection with sexual function [19,20].

Since this study focuses on the effect of weight loss and its link with improved sexual function, it highlights that often impact of obesity on sexual function is not discussed as one of its complications, which negatively affects a person’s quality of life. To the best of our knowledge, this is the first study in the region to explore the impact of weight loss in improving sexual function. However, the study has its limitation as well. First, since the study was conducted in a single institute, the sample size was less diverse. Secondly, socio-demographic factors were not considered, which might impact sexual satisfaction.

## Conclusions

Our study indicates that losing weight positively improves sexual function and has direct relation with percentage of weight lost. Based on our study, participants with high BMI should be counselled that obesity can impact their sexual function as well, which may cause in turn cause poor quality of life. It is important that participants with obesity should be counselled to lose weight to prevent various complications, including poor sexual function.
